# Effectiveness of Sotrovimab in the Omicron Storm Time: A Case Series

**DOI:** 10.3390/v15010102

**Published:** 2022-12-30

**Authors:** Gaetano Cicchitto, Lorena Cardillo, Davide Sequino, Paola Sabatini, Luisa Adamo, Rosita Marchitiello, Maurizio Viscardi, Loredana Cozzolino, Antonietta Cavallera, Marialuisa Bocchino, Alessandro Sanduzzi Zamparelli, Francesco Ferrigno, Esterina de Carlo, Claudio de Martinis, Giovanna Fusco

**Affiliations:** 1 COVID-19 Hospital “M. Scarlato”, Department of Pneumology, 84018 Salerno, Italy; 2 Istituto Zooprofilattico Sperimentale del Mezzogiorno, Via Salute 2, 80055 Naples, Italy; 3 Department of Clinical Medicine and Surgery, Section of Respiratory Disease Federico II University Hospital, 80055 Naples, Italy; 4 Umberto I” Hospital, Unit of Virology and Microbiology, Nocera Inferiore, 84018 Salerno, Italy; 5 COVID-19 Hospital “M. Scarlato”, Unit of Clinical Pathology Laboratory, 84018 Salerno, Italy; 6 COVID-19 Hospital “M. Scarlato”, Department of Radiology, 84018 Salerno, Italy

**Keywords:** SARS-CoV-2, monoclonal antibody treatment, sotrovimab, variant of concern, Omicron variant, immune suppression, chronic kidney disease

## Abstract

Neutralizing monoclonal antibodies (mAbs) for pre- and post-exposure prophylaxis of Severe Acute Respiratory Syndrome Coronavirus 2 (SARS-CoV-2) are largely used to prevent the progression of the disease by blocking viral attachment, host cell entry, and infectivity. Sotrovimab, like other available mAbs, has been developed against the receptor binding Domain of the *Spike* (S) glycoprotein of the virus. Nevertheless, the latest Omicron variant has shown marked mutations within the S gene, thus opening the question of the efficacy of these neutralizing molecules towards this novel variant. In the present observational study, we describe the effects of Sotrovimab in the treatment of 15 fully vaccinated patients, infected by SARS-CoV-2 Omicron sub-variants, who were selected on the basis of factors widely considered to affect a worse prognosis: immune suppression (*n* = 12) and/or chronic kidney disease (*n* = 5) with evidence of interstitial pneumonia in nine patients. The effectiveness of Sotrovimab in the treatment of severe cases of COVID-19 was demonstrated by the regression of symptoms (mean 5.7 days), no need of hospitalisation, improvement of general health conditions and viral clearance within 30 days in all patients. In conclusion, although loss or reduction of mAbs neutralizing activity against the Omicron variant have been described, Sotrovimab has clinically proven to be a safe and useful treatment for patients with high risk of progression to severe COVID-19 infected by Omicron sub-variants.

## 1. Introduction

More than 2 years after the onset of the Severe Acute Respiratory Syndrome Coronavirus 2 (SARS-CoV-2) pandemic, the morbidity and mortality of infected patients remain substantial, especially for those at risk of progression. Although mass vaccination programs have significantly diminished clinical burden and healthcare costs, the pandemic continues [1]. The reasons for this phenomenon can be traced back to variable individual behaviors, public health strategies, and the lack of completely effective treatments for SARS-CoV-2. As a consequence, the persistent spreading of the virus increases the potential for genome modification. The World Health Organization (WHO) and the Centers for Disease Control and Prevention (CDC), with the aim of proposing a shared working definition, identified changes in the genetic code of the virus and labeled these as variants of concern (VOC). These variants can cause an increase in transmissibility, a more severe disease, a significant reduction in neutralization by antibodies generated during previous infection or vaccination, a reduced effectiveness of treatments or vaccines or diagnostic detection failures [2,3].

Since the transmission of the infection usually occurs through an interaction between the viral spike (S) glycoprotein; a structural SARS-CoV-2 protein and a host cell receptor, such as the Angiotensin Converting Enzyme 2 (ACE 2); and the transmembrane serine protease type 2 (TMPRSS2) [4,5], the development of monoclonal antibodies (mAbs) targeting various epitopes of the S glycoprotein is considered an attractive therapeutic strategy. The Food and Drug Administration (FDA), the European Medical Agency (EMA) and the Italian Medicines Agency (AIFA), sequentially approved and/or authorized various mAbs for emergency use (EUA), individually or in combination, for the early treatment of COVID-19: bamlanivimab, bamlanivimab/etesevimab, casirivimab/imdevimab, sotrovimab and tixagevimab/cilgavimab [6,7,8]. Nevertheless, the emergence of the Omicron VOC caused the FDA to revise and limit the use of certain mAbs, mostly sotrovimab, only to patients that show susceptibility to these treatments due to the reduced neutralizing activity, in particular against the Omicron sub-variants [7,8]. However, most of the information on the efficacy of these neutralizing molecules is derived from in vitro studies and contrasts with real-world data on the efficacy of sotrovimab. Wu et al.—based on the evidence of their in vitro neutralization study, as well as real-world clinical efficacy data—suggest continuing the use of sotrovimab, especially in extremely vulnerable patients, given the absence of alternative and available treatment options [9].

In this paper, we describe the clinical outcomes of 15 fully vaccinated patients, who were infected by the recent Omicron VOC and treated with sotrovimab. They were selected on the basis of two strong anamnestic findings that are widely known to be responsible for a worse prognosis: immunosuppression, due to neoplastic or transplant related disorders, and chronic kidney diseases (CKD) [10,11].

## 2. Materials and Methods

### 2.1. Patient Enrollment, Clinical Data and Monoclonal Antibodies Administration

A total of 15 fully vaccinated and non-hospitalized patients, 7 males and 8 females, were enrolled from March to July 2022 in the “M. Scarlato” COVID-19 hospital (Scafati, Salerno, Southern Italy) for sotrovimab administration as they were considered at high risk of progression to severe COVID-19.

The eligibility criteria for the enrolment of the patients were based on the guidelines provided by the AIFA for the definition of the methods and conditions of use of the monoclonal antibody sotrovimab [11] as follows: (1) anamnesis of immunosuppressive disease and/or chronic kidney disease; (2) over 12 years of age; (3) at least 40 kg weight; (4) symptom onset within 7 days; (5) no need of supplement oxygen therapy or respiratory support; (6) presence of comorbidities. The degree of medical comorbidities was evaluated using Monoclonal Antibody Screening Score (MASS), as already described [12,13]. MASS was constructed by scoring individual diseases conforming to FDA and AIFA recommendations, designed to determine a clinical priority for treatment.

Symptoms’ burden reported by the patients on questionnaires was classified on the basis of the Center for Disease Control and Prevention (CDC) recommendations, as reported in previous research [14,15]. All patients underwent blood analyses, including interleukin 6 (IL-6), C-reactive protein (CRP), D-Dimer, and ferritin. As benchmarks of the inflammation, these parameters were tested to assess dysregulated systemic inflammation [16], as they are related to strong prognostic information. Moreover, all patients underwent high-resolution computed tomography (HRCT) to evaluate the pulmonary involvement, obtaining the total severity score (TSS). In particular, each of the five lung lobes was assessed for percentage of the lobar involvement and classified as none (0%), minimal (1–25%), mild (26–50%), moderate (51–75%), or severe (76–100%), with corresponding scores from 0 to 4. The TSS was obtained by summing the five lobe scores (range from 0 to 20) [17].

All the patients received 500 mg of sotrovimab (GlaxoSmithKline, Parma, Italy) intravenous (IV) infusion according to the guidelines reported by AIFA [11]. The administration of the drug was carried out within the hospital to allow for swift and appropriate management of any adverse event. Patients were clinically monitored for at least 1 h after the IV infusion, discharged and then managed as outpatients.

As indicated by AIFA, the follow-up was conducted for about 1 month after the administration of the mAb through remote contact (e.g., by telephone interview) with the patient on a weekly basis or, where necessary, by in-person presentation to the hospital, up to 30 days or until the achievement of symptom regression and negative results by real-time RT-PCR, and the end-of-treatment form was filled in [18]. The change in symptoms’ burden was quantified on the 7th day after the treatment and symptoms regression was intended with the improvement of general conditions. Moreover, nasopharyngeal swabs were carried out every 3 to 5 days, on the basis of symptoms and general clinical conditions, to assess the negativization of the patients.

Safety criteria were found in the reporting of adverse events, such as 72 h post-infusion-related reactions, changes in vital signs, or other circumstances, based on the Common Terminology Criteria for Adverse Events (CTCAE) [19].

### 2.2. Biomolecular Analysis

A multiplex real-time RT-PCR was carried out by the Unit of Virology and Microbiology of “Umberto I” Hospital using Allplex SARS-CoV-2 assay (Seegene, Seoul, Republic of Korea) that simultaneously detects 4 SARS-CoV-2 targets, RNA-dependent RNA polymerase (RdRP), Spike (S), Nucleocapsid (N) and Envelope (E) genes, following manufacturer’s instructions. Out of 15 nasopharyngeal swabs, 14 were then transferred in Universal Transport Medium (UTM) (Copan, Brescia, Italy) to the Istituto Zooprofilattico Sperimentale del Mezzogiorno Biosafety Level 3 (BLS-3) laboratory. The swabs underwent to nucleic acids extraction and purification using KingFisher Flex (Thermo Fisher Scientific, Waltham, MA, US) with an MVP_2Wash_200_Flex program following manufacturer’s instructions, eluted in a 50 µL final volume and stored at −80 °C until use. In BLS-2 laboratory, SARS-CoV-2 next-generation sequencing (NGS) was performed on Illumina MiSeq platform (Illumina, San Diego, CA), using MiSeqMicro Reagent Kit v2 (300-cycles) (Illumina), as described by the authors of a previous study [17].

The consensus sequences were obtained using the Geneious R9 software package (Biomatter) and submitted to GISAID database (http://www.gisaid.org accessed on 7 November 2022). The sequences are available under the following GISAID accession numbers: EPI_ISL_15303194; EPI_ISL_15303195; EPI_ISL_15303196; EPI_ISL_15303197; EPI_ISL_15303198; EPI_ISL_15303199; EPI_ISL_15303200; EPI_ISL_15303201; EPI_ISL_15303202; EPI_ISL_15303203; EPI_ISL_15303204; EPI_ISL_15303205; EPI_ISL_15303206; EPI_ISL_15303207.

## 3. Results

Out of the 14 sequenced samples, in eight patients, SARS-CoV-2 BA.5 Omicron sub-variants were revealed, followed by five BA.2 and 1 BA.1 sub-variants, confirming the exclusive circulation of the Omicron VOC and its sub-variants in the Salerno province (Campania Region, Southern Italy). In one patient (case 1), the NGS could not be performed, but he was SARS-CoV-2-positive during May 2022, a period in which the circulation in the Campania region was almost exclusively related to the Omicron variant, with an overall prevalence of 85% for the BA.2 variant, followed by the BA.5 and BA.1 variants: 8% and 1.3%; respectively [20].

Blood analyses showed that 14 patients had alterations in at least one biohumoral marker related to possible disease progression, even though severity levels were not reached. In addition, nine patients showed signs of interstitial pneumonia, although with minimal and mild lung involvement (HRCT-TSS < 50%) in the absence of respiratory failure. The anamnestic data show that 66.6% of the patients (*n* = 10) were immunosuppressed due to the use of immunosuppressive drugs: four patients for kidney transplants, for suffering from rheumatoid arthritis, two for Non-Hodgkin lymphoma, two with chronic lymphocytic leukaemia, and one because of aplastic anemia. Three more patients (20%) had CKD, and 13.3% (*n* = 2) suffered from a combination of immune-suppression and CKD. In the evaluation of the MASS, all patients showed further comorbidities, and cardiac diseases were the most frequently observed (*n* = 9).

Regarding vaccination prophylaxis, fifteen patients were fully vaccinated, and four patients received two doses vaccine. In two patients (case 8 and 13), a previous infection by SARS-CoV-2 was reported: ten patients were immunized with three doses and one patient received four doses. The results for signalment, anamnesis, blood and HRTC analyses, as well as biomolecular examinations, are reported in Table 1.

After sotrovimab infusion, patients were discharged, and ambulatorily monitored weekly for follow ups and to evaluate the onset of adverse events.

None of the patients needed hospitalization or died within 30 days post administration, and no side effects were observed or reported. Nevertheless, one patient progressed to mild respiratory failure within 24 h after infusion. He was treated as an outpatient, with low-flow oxygen and the best supportive therapy, quickly achieving an overall recovery of respiratory function.

Nasopharyngeal swab negative results by real-time RT-PCR were obtained between 3 and 24 days (mean 11.4 days) and symptoms regression occurred from 2 to 21 days after sotrovimab administration (mean 5.7 days).

## 4. Discussion

Although the implementation of mass vaccinations has remarkably reduced morbidity and mortality for COVID-19, the efficacy of vaccines is proving to wane over time, becoming suboptimal, mainly in immunocompromised individuals and those considered at high risk of severe disease [21,22]. Furthermore, as the global number of COVID-19 cases continues to increase, and the virus continues to mutate, the use of neutralizing monoclonal antibodies represents a promising option with therapeutic and prophylactic potential [23,24].

It is well known that the S glycoprotein promotes the viral entry into host cells, through the attachment of the SARS-CoV-2 receptor binding domain (RBD) to the human functional receptor ACE2. Therefore, the RBD has been used as the primary target for the development of currently approved mAbs [25,26]. Sotrovimab, a recombinant human IgG1-kappa mAb, was approved for emergency use in Italy in August 2021, suggested for use in patients with respiratory, cardiac, metabolic, and immunosuppression comorbidities [11]. The data available on the efficacy of this mAb are mainly based on in vitro analyses, but information on the in vivo activity of this neutralizing antibody is scarce and often contentious.

Currently, one of the most challenging aspects related to mAbs therapy concerns their efficacy, mainly associated with the emergence of new VOCs. Indeed, clinical trials conducted to support the use of mAbs were based on the enrolment of unvaccinated individuals and were performed during a period in which there were circulating variants that later disappeared, and thus before the spread of the most recent VOCs. Therefore, the conclusions of the efficacy of the approved mAbs in these trials are not applicable to patients in a modern-day scenario. Notably, the latest Omicron variant carries several mutations in RBD that determine the loss or reduction in SARS-CoV-2 susceptibility to the available mAbs [27,28]. This evidence has led international drug agencies to revise and limit the use of some monoclonal antibodies against the Omicron variant, encouraging healthcare providers to choose a therapeutic option on the basis of the circulating variant in their state [7,11]. Additionally, evolving sub-variant mutations of the same lineage could result in poor cross-reactivity and partial immune protection [29], causing individuals to repeatedly become infected [30], or risk the effectiveness of a treatment [31]. In a recent in vitro study, the lower neutralizing activity of sotrovimab against BA.1, BA.2 and BA.5 sub-lineages of the Omicron variant has been described, showing a 1.4-fold reduction against BA.2 compared to BA.1 and even a 2.7-fold-lower activity against BA.5 [28]; nevertheless, Izumo et al. [32], as well as Razonable et al. [13], observed low rates of severe disease after treatment with sotrovimab, demonstrating the high effectiveness of the molecule.

The present observational study aimed to evaluate the efficacy of sotrovimab in patients suffering from the immunosuppression of oncological diseases and chronic kidney diseases, two groups that are particularly vulnerable due to their association with a disrupt humoral response, and therefore considered seriously immunocompromised.

Although the clinical worsening of COVID-19 after the administration of mAbs has been reported—including fever, hypoxia or increased respiratory difficulty, arrhythmias, fatigue, etc. [7]—overall, our results show that the administration of sotrovimab seems to be safe in breakthrough SARS-CoV-2 infections in fragile patients, since no adverse effects occurred during infusion, post-infusion monitoring, and up to 30 days after infusion. Only one patient (case 1) progressed to mild respiratory failure and was treated as an outpatient with low-flow oxygen and the best supportive therapy. In this case, there were high viral loads (related to Ct value), a high comorbidity index, and only partial vaccination coverage, most likely due to ongoing immunosuppressive therapy. Altogether, these factors may have influenced the clinical course of the disease. Therefore, it cannot be excluded that the worsening of clinical conditions could also be ascribed to the underlying high-risk medical comorbidities [13] and not to the antibody-dependent enhancement of sotrovimab administration [33]. However, the patient did not require hospitalization, respiratory failure quickly regressed (within 2 days), and no complications were recorded at the 30-day follow-up; therefore, we could hypothesize a braking action of mAbs treatment also in this patient.

Additionally, we could highlight the safety and efficacy of sotrovimab therapy in this subgroup of vulnerable patients, since there were no hospitalizations, access to the emergency room, or deaths. It should be also underlined that all patients, except for one, displayed an alteration of at least one biohumoral marker that could be correlated to possible disease progression [17], even though the data did not reach particularly severe levels; in addition, many patients unveiled signs of interstitial pneumonia, but with less than a 50% lung involvement, in the absence of respiratory failure.

Regarding virological examinations, the average time elapsed for the negativization by real time RT-PCR on nasopharyngeal swab was 11 days, despite their immunocompromised status that is often associated with long-term viral shedding [34]. The possible association between prior infection and vaccination (“hybrid immunity”), could induce higher immune protection [35], helping in the clearance of the virus. On the other hand, we observed a reinfection in two previously vaccinated and infected patients, with (case 8) and without (case 13) pulmonary involvement, despite the supposed protective effects of this “hybrid immunity”. However, in both cases, there was no disease progression. Therefore, it is possible to hypothesize that the protective effects of both the active and passive immunological responses do not last particularly long. Thus, it is likewise difficult to interpret the role of the SARS-CoV-2 serum antibodies at baseline: they could reflect a vaccination status or a response to a natural infection and, in this regard, could express the absence of a protective effect or a restraining action on the patients’ clinical course. However, mAbs treatment among vaccinated subjects with breakthrough COVID-19 treatments seems to produce several clinical benefits, especially in multi-comorbidity outpatients [12]. Our study appears to confirm these data on the safety and effectiveness of treatment. There were no side effects of the therapy in all our patients, with variable levels of serum SARS-CoV-2 IgG anti spike antibodies, nor hospitalizations or access to the emergency room at 30 days of monitoring.

## 5. Conclusions

The emergence of the Omicron variant and its sub-lineages raised questions regarding the effectiveness of the approved therapy for early COVID-19. The available data on sotrovimab treatment are scarce, contentious, and often obtained from in vitro studies for the Omicron variant. In this “real life” cohort series of markedly vulnerable patients, sotrovimab therapy has proven to be clinically safe and useful for preventing disease progression in the Omicron sub-variant infection era. Furthermore, although a massive vaccination strategy has been established, the immune evasion of VOCs is still causing the spread of the virus, even with repeated infections. Our study highlights the importance of concentrating efforts not only on prevention but also on protection from the development of severe diseases, which constitute a health burden both in economic and social terms. These goals can be achieved through vaccination strategies and the early treatment (antiviral, mAbs) of selected patients.

## Figures and Tables

**Table 1:** Patient signalment, anamnesis and supplementary exam results.

Case	Age (y)	Smoke	Sex	MASS	Previous Infection (y/*n*)	Vaccination (*n*)	Last Vaccination (Months)	S.S.	Real-Time RT-PCR (Ct)	Detected Variant	Negative Swab (d)	From Onset to mAb Administration (d)	IgG Anti-S Antibodies (U/mL)	SaO2 (%)	IL-6 (pg/L)	CRP (mg/L)	D-dimer (ng/mL)	Ferritin (ng/mL)	HRCT TSS	S.R. (d)
1	80	NS	M	11	*n*	2	10	11	18	N/A	16	4	N/A	94	39.3	33.9	567	444	3	10
2	58	S	M	7	*n*	3	3	8	22	BA.5.2.1	7	4	8352	98	2.61	2.6	877	661	0	2
3	76	S	F	11	*n*	3	5	7	19	BA.5.1	8	2	N/A	97	11.6	13.2	340	244	4	4
4	47	S	M	4	*n*	3	3	12	24	BA.1.1.14	14	4	N/A	98	1.5	4.4	123	26	0	3
5	46	S	F	4	*n*	3	1	5	25	BA.2	9	4	N/A	97	22.3	7.1	NA	21	2	2
6	67	S	M	9	*n*	4	2	11	23	BA.2.9	18	3	40	98	10.5	5.9	360	147	2	3
7	70	NS	F	6	*n*	3	6	13	16	BA.2.3.15	7	4	92.6	98	3.0	1.1	475	28	3	21
8	82	NS	F	8	y	2	7	12	21	BA.5.1	24	4	2531	96	10.9	27.9	403	104	3	5
9	60	NS	F	4	*n*	2	6	14	21	BA.5.2	13	4	N/A	97	7.3	23.3	307	3536	2	2
10	59	NS	M	5	*n*	3	8	12	22	BA.5.1.10	11	3	6158	97	11	34.7	193	708	0	21
11	75	NS	M	6	*n*	3	7	10	20	BA.5.1	21	4	40	98	8.9	1.4	58	253	0	3
12	88	NS	F	6	*n*	3	9	9	22	BA.5.1	8	3	2757	97	26.1	37.8	270	471	0	2
13	67	FS	M	7	y	2	9	7	27	BA.5.2.1	7	4	7857	98	7	13.5	131	278	0	3
14	57	NS	F	9	*n*	3	6	7	17	BA.2.52	9	3	223	97	7.2	14.8	414	146	5	2
15	73	NS	F	6	*n*	3	6	7	28	BA.2	3	6	5000	95	19.5	1.1	504	647	2	3

**MASS**: Monoclonal Antibody Screening Score; **S.S.**: Symptom Score; **Ct**: cycle threshold; **SaO2**: hemoglobin oxygen saturation; **IL-6**: interleukin 6; **CRP**: C-reactive protein; **HRCT TSS**: High-Resolution Computed Tomography Total Severity Score; **S.R.**: symptoms reversion. **NS:** non-smoker; **S:** smoker; **FS:** former smoker. **N/A:** not assessed.

## Data Availability

Not applicable.
